# Developmental studies on the acquisition of perception conducting pathways via TRP channels in rat molar odontoblasts using immunohistochemistry and RT-qPCR

**DOI:** 10.1007/s12565-019-00517-y

**Published:** 2019-12-17

**Authors:** Aoi Tanaka, Yoshiyuki Shibukawa, Masahito Yamamoto, Shinichi Abe, Hitoshi Yamamoto, Seikou Shintani

**Affiliations:** 1 Department of Pediatric Dentistry, Tokyo Dental College, 2-9-18, Kanda Misaki-cho, Chiyoda-ku, Tokyo, 101-0061 Japan; 2 Department of Physiology, Tokyo Dental College, 2-9-18, Kanda Misaki-cho, Chiyoda-ku, Tokyo, 101-0061 Japan; 3 Department of Anatomy, Tokyo Dental College, 2-9-18, Kanda Misaki-cho, Chiyoda-ku, Tokyo, 101-0061 Japan; 4 Department of Histology and Developmental Biology, Tokyo Dental College, 2-9-18, Kanda Misaki-cho, Chiyoda-ku, Tokyo, 101-0061 Japan

**Keywords:** Immunohistochemistry, Odontoblast, PANX-1 channel, RT-qPCR, TRP channel

## Abstract

Odontoblasts act as dentin formation and sensory receptors. Recently, it was reported that transient receptor potential ankyrin (TRPA) 1, TRP vanilloid (TRPV) 4 and pannexin 1 (PANX-1) play important roles in odontoblast sensory reception. However, it is not known when odontoblasts begin to possess a sense reception function. The aim of this study was to clarify the development of odontoblasts as sense receptors. Sections of mandibular first molars from postnatal day (PN) 0 to PN12 Wistar rats were prepared for hematoxylin–eosin staining. Immunohistochemically, we used anti-dentin sialoprotein (DSP), anti-TRPA1, anti-TRPV4, anti-PANX-1, and anti-neurofilament (NF) antibodies. In addition, we investigated *TRPA1* and *TRPV4* expression by reverse transcriptional quantitative polymerase chain reaction (RT-qPCR). At PN0, undifferentiated odontoblasts showed no immunoreaction to anti-DSP, anti-TRPA1, anti-TRPV4, or anti-PANX-1 antibodies. However, immunopositive reactions of these antibodies increased during odontoblast differentiation at PN3 and PN6. An immunopositive reaction of the anti-NF antibody appeared in the odontoblast neighborhood at PN12, when the odontoblasts began to form root dentin, and this appeared later than that of the other antibodies. By RT-qPCR, expression of *TRPA1* at PN6 was significantly lower than that at PN0 (*p* < 0.05) and PN3 (*p* < 0.01). Expression of *TRPV4* at PN6 was significantly lower than that at PN0 (*p* < 0.01) and PN3 (*p* < 0.01). The results of this study suggest that odontoblasts may acquire sensory receptor function after beginning to form root dentin, when TRPA1, TRPV4, PANX-1 channels, and nerve fibers are completely formed.

## Introduction

Dental pulp, surrounded by hard tissue, is classified as loose connective tissue and is the only soft tissue among tooth components. In dental pulp, most cells are fibroblasts, and are referred to as dental pulp cells. There are also undifferentiated mesenchymal cells in dental pulp as in other loose connective tissue. However, odontoblasts are located only in dental pulp and are not found in other loose connective tissue. Odontoblasts originate from the ectomesenchyme derived from the neural crest to the maxillofacial area (Chai et al. 1998, 2000). Odontoblasts are differentiated from the dental papilla cells facing the inner enamel epithelium through epithelial-mesenchymal interaction and form dentin. An odontoblast has a long cell process in the dentinal tubule, referred to as Tomes’ fiber. Odontoblasts in dental pulp continue to survive throughout life unless damaged. This characteristic of odontoblasts differs from that of other cells except for some in the neural system (Nanci 2013).

Among the functions of dental pulp are dentin formation, sense reception and nutrition supply to dentin and so on. Of these, dentin formation is performed by odontoblasts. There are several theories regarding the sense reception of dental pulp: the hydrodynamic theory, the nerve endings theory and the odontoblast transducer theory. Although hydrodynamic theory is the most widely accepted theory, the sensitivity of the pulp reception mechanism remains unknown.

Transient receptor potential (TRP) channels are nonselective cation channels encoded in photoreceptor cells of the drosophila fly (Montell and Rubin 1989) and have been classified into six subfamilies (Minke 2002; Nilius and Voets 2005). These channels have various physiological functions, and their involvement in the sensing of chemical (capsaicin) and physical/mechanical (temperature, light, and pressure) stimulation has already been confirmed (Caterina et al. 1997; Clapham 2003; Hardie 2011; Uchida et al. 2017). It was reported that TRP channels were involved in the development, differentiation and maturation of the cells and tissues in salivary glands (Fujiseki et al. 2017), cerebellar neurons (Huang et al. 2007), and osteoclasts (Masuyama et al. 2008). Therefore, it is thought that TRP channels are multi-function channels. Pannexin (PANX) channels are classified as PANX-1, PANX-2, and PANX-3. PANXs channels are located on the plasma membrane, and are known to open following changes in intracellular calcium ion signaling, vasodilation, vasoconstriction, taste sensation, airway defense, learning/memory, cellular differentiation, cell death and adaptive immune responses (Chekeni et al. 2010; MacVicar and Thompson 2010). Small signaling molecules, such as ATP, are released into the extracellular space by the PANX-1 channels and ATP is incorporated into nerve fibers via P2X_3_, P2Y_1_, and P2Y_12_ receptors (Kawaguchi et al. 2015; Shibukawa et al. 2015).

It is known that TRP ankyrin (TRPA) 1 and TRP vanilloid (TRPV) 4 channels are expressed on odontoblasts (Sato et al. 2013; Egbuniwe et al. 2014; Kimura et al. 2016) Shibukawa et al. (2015). demonstrated that ATP, released from mechanically stimulated odontoblasts via pannexin-1 in response to TRP channel activation, upregulated P2X_3_ receptors on trigeminal (TG) neurons, increasing the intercellular calcium ions of the nerve. This suggests that mature odontoblasts play roles in both dentin formation and sense reception in terms of dental pulp functions. The odontoblast stage in which dentin formation occurred has been confirmed both in vitro and in vivo in various studies (Kjoelby et al. 1994; D’souza et al. 1997; Martinez et al. 2009; Tsujigawa et al., 2013; Sagomonyants and Mina 2014). However, it is not known when odontoblasts begin to act as sensory receptors.

We investigated the localization of the TRP and PANX-1 channels on odontoblasts and nerve fibers beneath the odontoblasts by immunohistochemistry in rat molars to clarify their development as sense receptors. In addition, we investigated expression levels of *TRP channels* during odontoblast differentiation by reverse transcriptional quantitative polymerase chain reaction (RT-qPCR).

## Materials and methods

This study was approved by the Tokyo Dental College Experimental Animal Committee and conformed with the specified guidelines for animal experiments (No. 292,302).

### Histology and immunohistochemistry

Twenty-five Male Wistar rats at postnatal day (PN) 0, 3, 6, 9, and 12 (five per stage) were used for histological and immunohistochemical analyses. Rats were deeply anesthetized with isoflurane (3vol%) and intraperitoneal injection of pentobarbital (30 mg/kg). Rats were fixed by perfusion of 0.1 M phosphate buffered saline (PBS) buffered in 4% paraformaldehyde solution (pH 7.4). Then, the mandible including the first molar was removed and immersed in fixation fluid at 4 °C for 24 h. The mandible was decalcified with 10% EDTA at 4 °C for 3–4 weeks. After washing with PBS, dehydration with ethanol series was carried out. Then specimens at PN 0, 3 and 6 were embedded in paraffin by a conventional method. For frozen sections, some specimens were immersed in 10%, 20%, and 30% sucrose in PBS at PN 9 and 12 after decalcification, and then embedded in O. C. T. Compound (Sakura Finetek USA, Inc., CA, USA). Thick serial sections were prepared (paraffin section: 4 µm. frozen section: 40 µm). Standard hematoxylin–eosin double staining was applied. Some sections were subjected to immunohistochemical staining as follows: Sections were deparaffinized with xylene and an alcohol series or were washed with PBS, then immersed in methanol containing 0.3% hydrogen peroxide (H_2_O_2_) at room temperature for 30 min to remove endogenous peroxidase. Then, the sections were blocked with 2.5% goat serum. Immunostaining was performed using the VECTASTAIN Elite ABC Kit (Vector Laboratories, Inc., California, USA) with the following primary antibodies: A rabbit anti-rat dentin sialoprotein (DSP) polyclonal antibody (1/500, Santa Cruz Biotechnology, Texas, USA), a rabbit anti-rat TRPA1 polyclonal antibody (1/1000, Abcam, Cambridge, UK), a rabbit anti-rat TRPV4 polyclonal antibody (1/500, Abcam, Cambridge, UK), and a rabbit anti-rat PANX-1 polyclonal antibody (1/400, Cosmo bio, Inc., Tokyo, Japan) were used in the paraffin sections. A rabbit anti-rat 200 kD neurofilament heavy (NF) polyclonal antibody (1/500, Abcam, Cambridge, UK) was used in the frozen sections, and the dark brown color was developed using 3,3′-diaminobenzidine tetrahydrochloride, followed by counter staining with hematoxylin. The sections were reacted with normal rabbit serum instead of the primary antibody as a negative control.

### RT-qPCR

Mandibular first molar tooth germs were extracted from rats immediately after sacrifice under deep anesthesia in the same way as for histology and immunohistochemistry. Enamel organ and dental papilla were separated mechanically and only the dental papilla was immersed into an RNA*later* RNA Stabilization Reagent (QIAGEN, Limburg, Germany). Total RNA was extracted from dental papilla with an RNeasy Micro Kit (QIAGEN, Limburg, Germany) according to the manufacturer’s instructions, and 1 µg of RNA was reverse-transcribed into cDNA using a QuantiTect Reverse Transcription Kit (QIAGEN, Limburg, Germany). The reaction mixture was added to the RNA solution and incubated at 42 °C for 15 min to synthesize cDNA, followed by incubation at 95 °C for 3 min to inactivate the enzymes. Real-time PCR was performed using Premix Ex Taq™ (Perfect Real Time) (TaKaRa Bio, Inc., Shiga, Japan) and an Applied Biosystems 7500 Fast Real-Time PCR System (Thermo Fisher Scientific, Massachusetts, USA). Specific primers for rats and the Universal Probe Library (UPL) are shown in Table 1. Real-time PCR conditions were as follows: Enzyme activation, 95 °C for 30 s; amplification process, 95 °C for 3 s, 60 °C for 30 s; number of cycles, 40. Each mRNA expression level relative to the 18S rRNA mRNA expression level in the sample was determined using the 2(-ΔΔCT) method.

**Table 1:** The base sequences of the primers in RT-qPCR

	Primer	Sequence	UPL
*TRPV4*	Forward primer	CACAAGAAAGCCGATATGAGG	#49
	Reverse primer	GGGAGCACTTGAGAAGCAAC	
*TRPA1*	Forward primer	GGACCTCACTTCTCTTTTGGA	#94
	Reverse primer	TCCTTTTTGAACCTGTCTTGG	
*18S rRNA*	Forward primer	GGTGCATGGCCGTTCTTA	#22
	Reverse primer	AACTAGTTAGCATGCCGAGTC	

### Statistical analysis

The significance of differences was assessed using the Steel—Dwass test of EZR version 1.37.

## Results

### Histology and immunohistochemistry

**Fig. 1:**
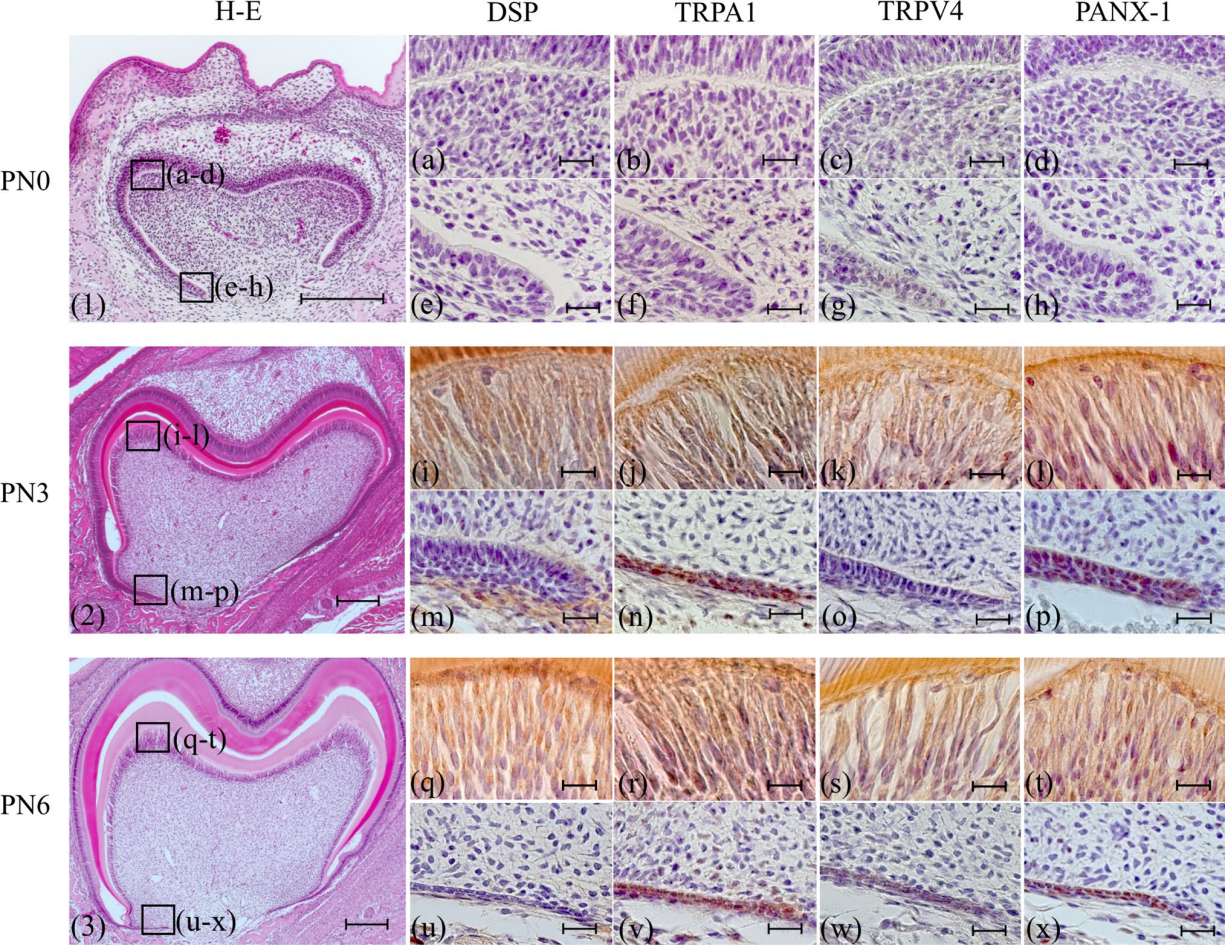
Histology (1–3) and immunohistochemistry (**a**–**x**). (1, **a**–**h**): PN0, (2, **i**–**p**): PN3, (3, **q**–**x**): PN6. (1)–(3): H-E staining, (**a**), (**e**), (**i**), (**m**), (**q**), (**u**): anti-DSP antibody, (**b**), (**f**), (**j**), (**n**), (**r**), (**v**): anti-TRPA1 antibody, (**c**), (**g**), (**k**), (**o**), (**s**), (**w**): anti-TRPV4 antibody, (**d**), (**h**), (**l**), (**p**), (**t**), (**x**): anti-PANX-1 antibody. (**a**–**d**), (**i**–**l**), (**q**–**t**): higher magnifications of the boxed area in the pulp horn region in (1–3). (**e–h**), (**m**–**p**), (**u**–**x**): higher magnifications of the boxed area in the cervical region in (1–3). At PN0, dental papilla cells (DP) did not differentiate into odontoblasts (1). No immunoreaction was observed in either the pulp horn or cervical region (**a**–**h**). After PN3, DP in the pulp horn differentiated into odontoblasts, although DP in the cervical region did not (2, 3). In the pulp horn region, odontoblasts showed immunoreactivity for anti-DSP, anti-TRPA1, anti-TRPV4 and anti-PANX-1 antibodies, although DP did not react immunohistochemically to these antibodies in the cervical region (**m**–**p**, **u**–**x**). Immunopositive reaction of anti-TRPA1 antibody was observed in HERS (**n**, **v**). Bars: (1, 2, 3) 200 µm, (**a–x**) 20 µm

**Fig. 2:**
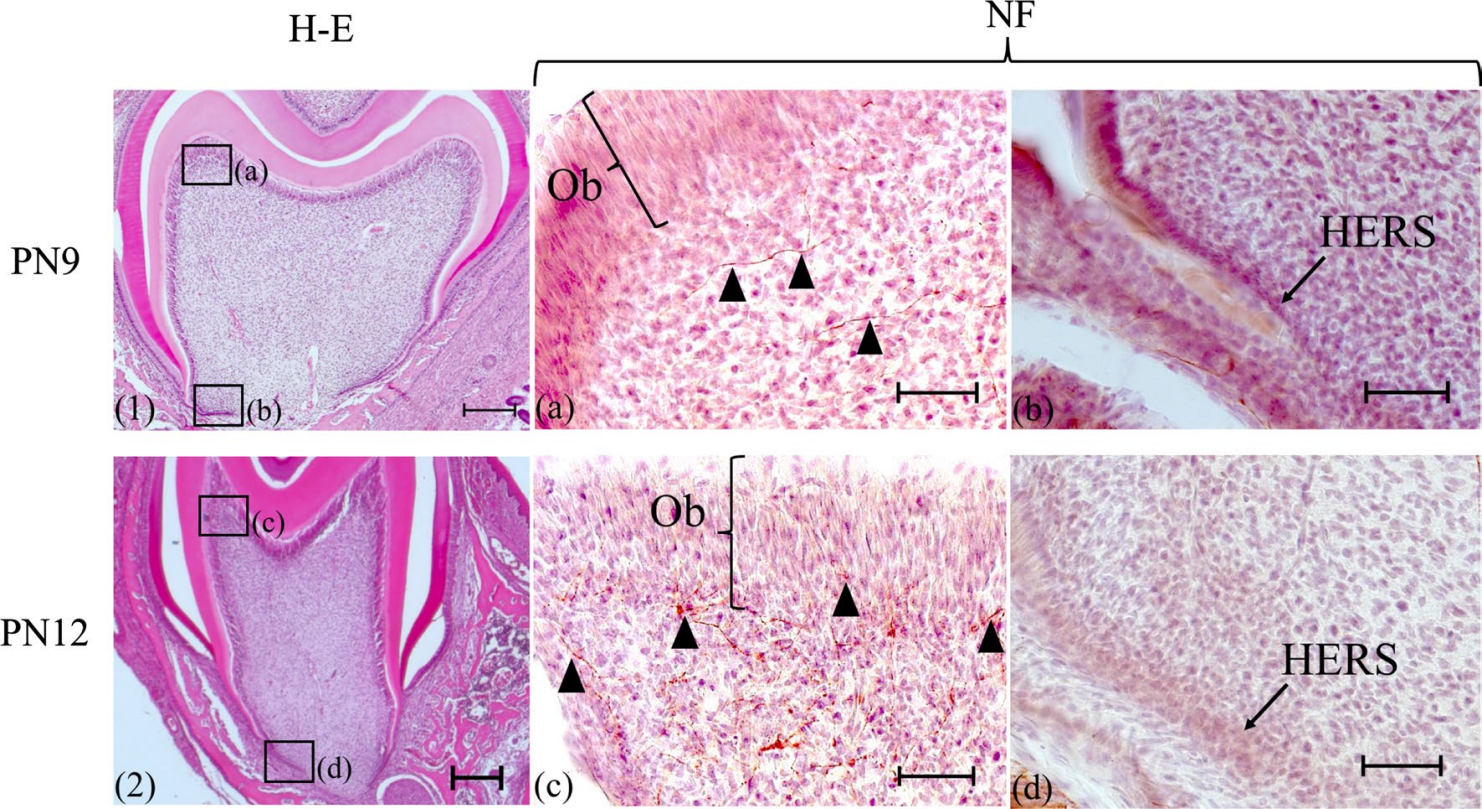
Histology (1, 2) and immunohistochemistry (**a**–**d**). (1, **a**, **b**): PN9, (2, **c**, **d**): PN12. (**a**–**d**): anti-NF antibody. **a**, **c** Higher magnifications of the boxed area in the pulp horn region in (1,2). **b**, **d** Higher magnifications of the boxed area in the cervical region in (1, 2). In the pulp horn region, an immunopositive reaction for an anti-NF antibody was observed slightly distant from the odontoblast layer in the pulp at PN9 (A arrowheads), while an immunoreaction was shown in and beneath the odontoblast layer at PN12 (**c** arrowheads). On the other hand, dental pulp cells facing Hertwig’s epithelial root sheath did not react with the anti-NF antibody (**b**, **d**). Ob: Odontoblast layer, HERS: Hertwig’s epithelial root sheath. Bars: (1, 2) 200 µm, (**a**–**d**) 50 µm

**Fig. 3:**
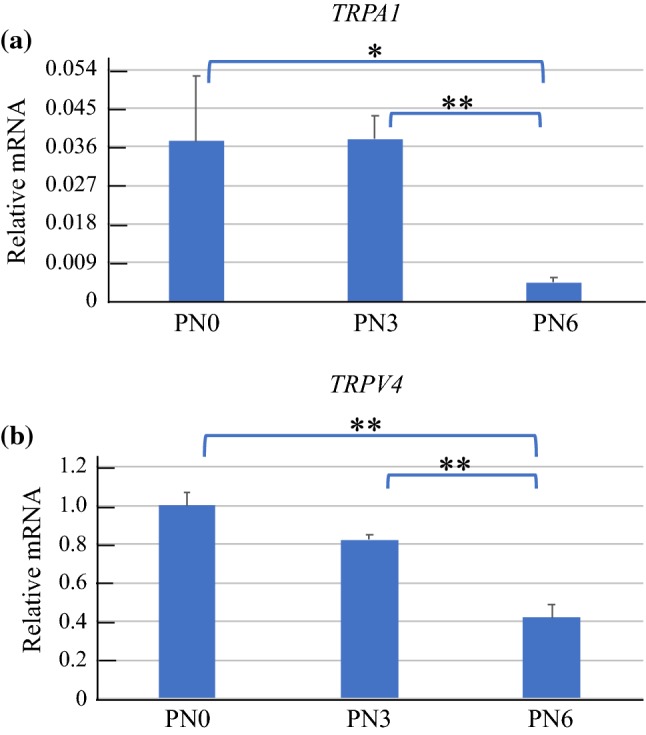
**a** Real-time PCR TRPA1 Results. Although expression of *TRPA1* at PN6 was significantly lower than at PN0 (*p < 0.05) and PN3 (**p < 0.01), there was no significant difference between PN0 and PN3. **b** Real-time PCR TRPV4 Results. Although expression of *TRPV4* at PN6 was significantly lower than at PN0 (**p < 0.01) and PN3 (**p < 0.01), there was no significant difference between PN0 and PN3

At PN0, dental papilla cells facing the inner enamel epithelium were a cuboidal shape in the pulp horn and cervical regions (Fig. 1-1). At this time point, dentin was not formed (Fig. 1-1). Immunoreactions for anti-DSP, anti-TRPA1, anti-TRPV4 and anti-PANX-1 antibodies were not observed in dental papilla cells facing the inner enamel epithelium (Fig. 1a–h). At PN3 and PN6, dental papilla cells facing the inner enamel epithelium differentiated into odontoblasts and dentin was formed in the pulp horn region (Figs. 1, 2, 3). Immunopositive reactions for anti-DSP, anti-TRPA1, anti-TRPV4 and anti-PANX-1 antibodies were observed in columnar odontoblasts in the pulp horn region (Fig. 1I, l, q–t). However, in the cervical region, dental papilla cells did not differentiate into odontoblasts and no immunoreaction was found (Fig. 1m–p, u–x). Immunopositive reaction of anti-TRPA1 antibody was observed in HERS. At PN9, the odontoblasts still formed coronal dentin (Fig. 2-1). On the other hand, the odontoblasts had already finished forming coronal dentin at PN12, and began to form root dentin (Fig. 2-2). A string-formed immunopositive reaction for an anti-NF antibody was observed in a region near the pulpal center separate from odontoblasts in the pulp horn region at PN9 (Fig. 2a), and an immunoreaction for an anti-NF antibody was observed in the odontoblast neighborhood at PN12 (Fig. 2c). No immunoreaction was found in the cervical region at PN9 and PN12 (Fig. 2b, d). Immunoreaction with anti-NF did not be found at PN6 (data not shown).

### RT-qPCR

Figure 3 shows the expression level of *TRPA1* and *TRPV4* based on that of *18S rRNA*.

Although expression of *TRPA1* at PN6 was significantly lower than that at PN0 (*p* < 0.05) and PN3 (*p* < 0.01), there was no significant difference between PN0 and PN3 (Fig. 3a). Expression of *TRPV4* at PN6 was significantly lower than that at PN0 (*p* < 0.01) and PN3 (*p* < 0.01), but there was no significant difference between PN0 and PN3 (Fig. 3b). The expression levels of *TRPA1* and *TRPV4* decreased with age.

## Discussion

A sense pathway is established as follows. First, a receptor receives stimulation. Then, the receptor releases a neurotransmitter, and finally nerve fibers are stimulated by the neurotransmitter. Therefore, the sense pathway requires receptors, neurotransmitters from the receptors, and nerve fibers. We immunohistochemically investigated the receptors, the TRPA1 and TRPV4 channels that receive stimulation, and the PANX-1 channels that release ATP as a neurotransmitter from odontoblasts (Shibukawa et al. 2015), using antibodies of these channels and nerve fibers during odontoblast differentiation. In addition, the state of odontoblast differentiation was examined by an anti-DSP antibody. As a result, it was found that the dental papilla cells, which are undifferentiated odontoblasts, facing the inner enamel epithelium did not react with anti-DSP, anti-TRPA1, anti-TRPV4, or anti-PANX-1 antibodies. However, the odontoblasts, which formed dentin and reacted immunoreactively with the anti-DSP antibody, showed immunopositive reactions with each antibody. This suggests that although TRPA1, TRPV4, and PANX-1 channels are not present in undifferentiated odontoblasts, these channels are expressed in odontoblasts that form the dentin matrix. This result was found that HERS reacted with anti-TRPA1 antibody. It is known that TRPA1 channel is expressed on epithelium (Atoyan et al. 2009). HERS is constructed by inner enamel epithelium and outer enamel epithelium, thus TRPA1 channel is expressed on HERS. On the other hand, previous immunohistochemical studies also reported that nerve fibers near odontoblasts were observed around PN12 (Corpron and Avery 1973; Fristad et al. 1994; Veerayutthwilai et al. 2006). In the present study, an immunopositive reaction with an anti-NF antibody beneath the odontoblast layer was observed in dental pulp at PN12. Based on our results and previous research, it is suggested that the TRPA1 and TRPV4 channels of odontoblasts may play a role as sensory receptors around PN12 when the odontoblasts have already finished forming coronal dentin and have started to form root dentin.

In our RT-qPCR study, the mRNA level of *TRPA1* was high at the onset of protein detection, and *TRPV4* before protein detection was higher than that after protein detection. Sato et al. (2013) and Kimura et al. (2016) suggested that TRPV4 and TRPA1 channels are involved in dentin formation, because immunoreactions of anti-TRPA1 and anti-TRPV4 antibodies were observed in odontoblasts that formed the dentin matrix. However, the mRNA of *TRPA1* and *TRPV4* gradually decreased. It is considered that tooth germs get bigger with age each day. The ratio of undifferentiated odontoblasts decreased in tooth germs. The mRNA of TRPA1 and TRPV4 were high on the undifferentiated odontoblasts, although the mRNA decreased in our RT-qPCR study.

It is interesting that the sense pathway via odontoblasts is established before tooth eruption. After tooth eruption, the teeth are stimulated by various stresses such as bacterial and mechanical stimulation. To sense pain is an important defense mechanism for the body. The sense pathway via odontoblasts is established before tooth eruption, suggesting that it is a defense mechanism against various stresses after tooth eruption.

In conclusion, we revealed that teeth at PN12 already expressed all of TRPA1 channel, TRPV4 channel, PANX-1 channel, and nerve fibers, although nerve fibers did not reach odontoblasts at PN9. These findings suggest that odontoblasts may acquire a sensory receptor function, because TRPA1, TRPV4, PANX-1 channels and nerve fibers are completed when the odontoblasts begin to form root dentin.
